# A genome-scale metabolic model of the lipid-accumulating yeast *Yarrowia lipolytica*

**DOI:** 10.1186/1752-0509-6-35

**Published:** 2012-05-04

**Authors:** Nicolas Loira, Thierry Dulermo, Jean-Marc Nicaud, David James Sherman

**Affiliations:** 1 Inria / Université Bordeaux / CNRS joint project-team MAGNOME, Talence, F-33405, France; 2 INRA, UMR1319 Micalis, Jouy-en-Josas, F-78352, France; 3 CNRS, Micalis, Jouy-en-Josas, F-78352, France; 4 Center for Genome Regulation, Universidad de Chile, Av. Blanco Encalada 2085, 3er piso, Santiago, Chile

## Abstract

**Background:**

*Yarrowia lipolytica* is an oleaginous yeast which has emerged as an important microorganism for several biotechnological processes, such as the production of organic acids, lipases and proteases. It is also considered a good candidate for single-cell oil production. Although some of its metabolic pathways are well studied, its metabolic engineering is hindered by the lack of a genome-scale model that integrates the current knowledge about its metabolism.

**Results:**

Combining *in silico* tools and expert manual curation, we have produced an accurate genome-scale metabolic model for *Y. lipolytica*. Using a scaffold derived from a functional metabolic model of the well-studied but phylogenetically distant yeast *S. cerevisiae*, we mapped conserved reactions, rewrote gene associations, added species-specific reactions and inserted specialized copies of scaffold reactions to account for species-specific expansion of protein families. We used physiological measures obtained under lab conditions to validate our predictions.

**Conclusions:**

*Y. lipolytica* iNL895 represents the first well-annotated metabolic model of an oleaginous yeast, providing a base for future metabolic improvement, and a starting point for the metabolic reconstruction of other species in the *Yarrowia* clade and other oleaginous yeasts.

## Background

Even if lipid metabolism is common to all microorganisms, we call *oleaginous* those that can store at least 20% of their dry mass as lipids. It is possible to find oleaginous organisms among plants, algae, bacteria and yeasts. Plants and algae are technically difficult (and controversial) to modify genetically, while oleaginous bateria present a low growth rate. On the other side, oleaginous yeasts enjoy well-developed genetic tools for their improvement and grow quickly. Also, oleaginous yeasts can accumulate up to 70% of their dry mass as lipids [1], making them the best candidates for industrial lipid production such as microbial oil for biodiesel.

One of those oleaginous yeasts, *Yarrowia lipolytica*, normally found as a food contaminant, has been extensively studied experimentally. It is easy to modify genetically, and presents many opportunities for metabolic engineering. For example, *Y. lipolytica* has been used as a food supplement, given its easily modifiable lipid composition. It is also studied as a potential source of biodiesel [2-4], because lipids producedby this species are similar to vegetable oils and fats. While *Y. lipolytica* is a hemiascomycete yeast, it is phylogenetically distant from *S. cerevisiae* and other well-studied yeasts, manifesting many metabolic differences: it is an obligate aerobic yeast, that can use normal hydrocarbons and various fats as carbon sources; it secretes diverse hydrolytic enzymes (proteases, lipases, RNases); its perixosome is constitutive.

Metabolic models are an important tool for metabolic engineering. Their uses include the guidance of metabolic engineering, the contextualization of high-throughput data and helping hypothesis-driven discovery.

Genome-scale metabolic models have up to now been principally produced for bacterial species and for a few higher organisms (see [5] for a review). This focus on model organisms is in part due to the great cost of obtaining high-quality annotated complete genome sequences, which requires considerable human effort regardless of the relative low cost of obtaining the genome sequence. A further need is to produce new experimental data to verify and improve the reconstructed model. Most models are reconstructed starting from the genome annotation, assembling known reactions into connected networks [6]. This requires a lengthy and expensive period of manual curation. Software has been designed to deal with process, although most existing tools are designed for bacteria.

*Y. lipolytica* is an ideal species for metabolic reconstruction in eukaryotes through comparative genomics. As one of the hemiascomycetous yeasts completely sequenced in the Génolevures program, it enjoys a high quality manual annotation by a network of expert curators [7,8]. Careful analysis of conservation and species-specific expansion and contraction of families of protein-coding genes makes it possible to identify orthologs with known genes in the clade as well as functionally important paralogous families. The conservation of core metabolism with other yeasts is enough to allow the use of existing metabolic models from *S. cerevisiae* as a template, into which species-specific reactions and secondary metabolism can be assembled.

In this work we present the first genome-scale functional metabolic model for *Y. lipolytica*, built with an iterative process of automatic reconstruction and manual curation. We started from a scaffold derived from existing *S. cerevisiae* models, extracting information about enzymatic reactions, molecular species, transport reactions, and compartments. With this scaffold we built an *in silico* draft by mapping known enzyme-encoding genes, using gene homology information obtained from Génolevures protein families [8,9] and complemented with other *in silico* methods, and filled network gaps in order to make it functional (i.e. to be able to predict growth from available metabolites in the media). We performed a manual curation of the initial draft model, adding species-specific metabolic reactions, in particular those related with central carbon and fatty acid metabolism. To assess the predictive power of our model, we compared our predictions against published experimental results of growth under different media conditions and gene knockouts. This comparison shows high degree of agreement between predictions and experimental results.

## Results

### Properties of the model

Our functional genome-scale metabolic model for *Yarrowia lipolytica* iNL895 describes 2 002 reactions encoded by 895 *Y. lipolytica* genes, the 1 847 metabolites consumed and produced by those reactions, the 16 compartments in which those reactions take place and a biomass function which describes the metabolic requirements for growth.

From the total of reactions, 139 (7%) are transport reactions with a gene association, 286 (14.3%) transport reactions that are spontaneous or without a known gene association, 171 (8.5%) are exchanges with the media, 1 055 (52.7%) enzymatic reactions with a gene association and 351 (17.5%) without.

The 1 055 enzymatic reactions with associated genes in the curated model were distributed into 39 biological processes, based on the associated GO Slim annotation of the closest ortholog in *S. cerevisiae.*

#### Gains

For alkane degradation we have introduced the ω-oxidation pathway including cytochrome P450 oxidases (12 genes ALK1–ALK12) and the cytochrome P450 reductase (CPR) reaction from each alkane (decane, dodecane, hexadecane, etc.) to the corresponding alcohol. We added reactions from alcohols to aldehydes then aldehydes to corresponding fatty acids, following [10].

For triglyceride degradation *Y. lipolytica* secretes lipases that are either extracellular (Lip2p) or membrane-bound (Lip7 and Lip8). These are part of a 19-gene multi-gene family [3].

For fatty acid synthesis we have included in reaction r_2008 the effect of a new gene that codes a member of the type 1 acyl-CoA:diacylglycerol acyltransferase family (DGAT1), which has not previously been identified in yeasts, but is commonly found in mammals and plants, and proposed in [11] to give the oleaginous character.

For transport and export of hydrophobic substrates we have included reactions for binding, export (by an ABC transporter, the gene ABC1 for alkane utilization [10]), and metabolite transport. The latter transport is necessary to explain growth of the TCA cycle mutant and the malate dehydrogenase mutant..

#### Losses

For oleaginous character, we have taken into account the loss in *Y. lipolytica *of genes linked to glycerol 3 phosphate (G3P), following [12], specifically in reaction r_0528 reported in Additional file 1: Table S1.

For galactose and sucrose substrates we verified the absence of reactions that are not present in *Y. lipolytica* due to missing genes (compared to *S. cerevisiae*), in particular *Y. lipolytica* cannot use sucrose as a sole carbon source due to the lack of the corresponding invertase. Note that transformation of *Y. lipolytica* strains is made possible through the inclusion of a selective marker built from a fusion of the *S. cerevisiae* SUC2 gene with the promoter and signal sequence of *Y. lipolytica* alkaline extracellular protease XPR2 [13].

For ethanol production, unlike *S. cerevisiae*, *Y. lipolytica *uses ethanol only with difficulty and does not produce it. These losses are reflected in reactions r_0176 through r_0190 concerning acetaldehyde to ethanol transformation.

### Validation of the model

The draft model was verified by experts in *Y. lipolytica*, and approved in terms of agreement with the literature: This model is not capable of producing ethanol, it cannot grow anaerobically, fatty acid metabolism presented expansions and contractions of protein families, and new species-specific reactions for the intake of alkanes were automatically detected.

Also, to assess the completeness of our model, we compared its phenotypic predictions in terms of growth/no growth, against published experimental results of observed growth, under several carbon sources and gene knockouts ( Additional file 2: Table S2). We used flux balance analysis (FBA), and a constraint based optimization approach [14] to predict whether a phenotype was present. After defining restrictions in the intake capacity of the organism, based on a selection of experimental data, we used FBA to predict biomass production, and thus the capacity of the organism to grow under those restrictions. Gene knockouts were modeled as deletions in the reconstructed metabolic network.

Media conditions, in particular different carbon sources, were extracted from the literature (See Table 1). Alas, not all experiments were well documented in terms of molecular species present in the media, so a rich media (YPD) was assumed and modified based on the general description of the media. See [15] for a discussion about uncertainty in media conditions.

**Table 1:** Experimental conditions used for validation

Reference	Gene KOs	Media conditions
BioloMICS [16]	–	46 different carbon sources
Thevenieau,2007 [10]	15 gene KOs	YNBD, YNBO, YNBC10, YNBC16, YNBT
T van den Temple, 2000 [17]	–	Lactose, D-Galactose
Jardon, 2008 [18]	*FBP1*	YNBD, Ethanol, Glycerol, Acetate
Flores, 2005 [19]	*PYC1, ICL1*	YNBD, Ethanol, Aspartate, Glutamate
Yamagami, 2001 [20]	*PAT1*	YNBC10, YNBD, Glycerol
Haddouche (PC) [21]	*ACL1*	YNBD, YNBO
Kabran, 2010 [22]	*ICL1, MLS1, CIT2*	Acetate, YNBO, YNBD
Beopoulos, 2008 [23]	*GUT2, POX1-6*	YNBD, Glycerol, YNBO
Jiménez-Bremont, 2001 [24]	*OCD1*	YNBD, YNBD + putrescine
Cheon, 2003 [25]	*TRP1*	YNBD, YNBD + tryptophane

Literature sources used for validation of the *Y. lipolytica* model. Overall, 60 different media conditions were tested. Gene knockouts were assessed for 29 different *Y. lipolytica* gene loci, in 152different experiments. Only those cases where evident growth/no growth was observed were included in this analysis.

In order to facilitate comparison, quantitative results from experiments and from simulations of biomass production were simplified into binary values (growth/no growth). Corresponding binary results were obtained for 98 experiments paired with simulations, with exact agreement in 64 cases (39 true positives and 25 true negatives). The 18 false negatives we observed may be attributed to missing reactions, corresponding to *Y. lipolytica *genes that are still unannotated, or to gaps in understanding of redundancy in the network. These 18 cases are currently being used to target improvements in gene annotation. The remaining cases, 16 false positives, are likely the product of over-optimistic flux simulations and can be reduced through parameter tuning. Overall, using this simplified binary comparison we obtain an accuracy (geometric mean of sensitivity and specificity) of 0.65.

We stress that this qualitative validation does not substitute for quantitative comparison, but does show that each of the tested conditions is connected from uptake through to the biomass function. It thus serves to validate the completeness of the model, in particular with respect to overall network topology.

## Conclusions and discussion

Combining *in silico* tools and expert manual curation, we produced an accurate genome-scale metabolic model of the oleaginous yeast *Y. lipolytica*, using a functional metabolic model of the phylogenetically related yeast *S. cerevisiae* as a scaffold for the reconstruction. The method developed in the present work can be used for *genome-scale metabolic model* reconstruction of other organisms, making it a useful tool for biotechnology and research.

We noticed that, even if the list of *S. cerevisiae* reactions not present in *Y. lipolytica* was short, there was an important number of changes in the gene associations between both organisms. Also, the loss of some phenotypes in *Y. lipolytica*, compared to *S. cerevisiae*, was characterized by a loss of a small number of genes.

Thirteen new transport reactions were added to the new model in order to connect enzymatic reactions inside the peroxisome with molecular species in the cytosol, and to import species from extracellular space to the cytosol. We could not find genes encoding for all those transports, but we expect that the eventual characterization of the 1 034 (16%) *Y. lipolytica* genes with unknown function, will provide evidence for some of them. The lack of accuracy at predicting some experiments could be explained by missing reactions in the model, especially regarding the transport of specific carbon sources. This gives us hints about possible ways to improve our model.

The modifications to the draft model performed by the manual curators allowed us to formalize a set of edit operations over metabolic models. This facilitated an automatic iteration process, from improvements to the reconstruction method, to improved draft models, to automatic application of curator edits, to automatic assertion of accuracy.

The present model can be used to predict growth under different media conditions and gene knock-outs. It can also be used as a general description of the state-of-the-art in *Y. lipolytica* metabolism. Data from high-throughput experiments, like microarrays and metabolomics, can be mapped to this model to have an overview of metabolic changes under different media conditions.

Current understanding of *Y. lipolytica* is constantly improving, and a number of features of its metabolism are the subject of ongoing work and consequently improvements to the model. Multigene families such as POX1–POX6 in peroxisomal β-oxidation could be modeled with better precision, since there are enzymatic specificities linked to the length of the carbon chain (e.g. Pox2 for long chains, Pox3 for short chain fatty acids, see for example [26]). This is also true for multigene families LIP1–LIP19 hydrolases of triacylglycerides, where there also exists chain-length specificity [3], although the specificities of the ALK1–ALK19 genes are not completely known. In general, lipid metabolism in *Y. lipolytica* is still under study and there is a lack of knowledge in several areas, such as transport between compartments, or the link between nitrogen abundance and the production of either lipid or citric acid [11].

Expansion of families of isozymes is detectable through expansion of paralogous protein families, but the method used here cannot detect these differences because FBA does not differentiate isoenzyme activities in the same reaction. Dynamic models that describe the kinetics of individual enzymes in reactions must be developed. This will require acquiring and integrating metabolic and transcriptomic data for targeted pathways, and developing models. Alvarez-Vasquez *et al.*[27], for example, used biochemical systems theory to develop a model of *S. cerevisiae* sphingolipid metabolism; more recently, Gupta *et al.*[28] developed a quantitative model of this pathway in mammalian cells by combining metabolite and transcriptome data in their estimation of kinetic rate constants. In general, the constraint-based FBA approach used here for validation cannot describe *Y. lipolytica* metabolic pathways with the same precision as dynamic differential equation models, but does have the merit of permitting a whole-genome model.

The most pressing need in further iterations of the model is refinement of alkane degradation for decane and hexadecane. Indeed the analysis of alkane growth of ANT1 and ABC1 mutants were performed on n-alkane from C10 to C16, including C11, C13, and C15, in [10]. Also, *Y. lipolytica* is described as growing on n-alkane paraffin (petroleum distillate) containing n-alkane oil (C12 to C18 n-akanes) and also n-paraffin wax (C20 and above, solid alkane) in [29]. This suggests that it is necessary to introduce all even and odd chain lengths including C1, since *Y. lipolytica* could use very long alkane chains above C20.

## Methods

### Scaffold-based reconstruction

Genome-scale metabolic models describe the network of enzymatic and transport reactions in an organism. The main idea of most metabolic model reconstruction algorithms is to look for the presence of enzymatic reactions in the annotated genome of the organism to be modeled, and create a network of those reactions, representing the interconnected production and consumption of metabolites [6].

The construction of metabolic models is costly and time consuming, so tools have been developed to automatically create initial, draft versions of the models, to be further improved by manual curation. Some of the current methods and platforms are *Pathway Tools*[30], The SEED [31], AUTOGRAPH [32], and several machine learning methods [33].

These methods are mostly designed for bacterial organisms and are not always adequate for reconstruction of yeasts models. In particular, some of them lack proper handling of compartments, rewriting of gene associations, or rely on the strong functional relations provided by operons. Also, fine tuning existing programs was not always possible, given the lack of public source code availability. To cover these shortcomings, we implemented our own automatic reconstruction method (to be published separately). See Additional file 3: Figure S1 for an overview of our method.

Briefly, the method developed for the present work uses a scaffold model for the reconstruction. For each one of the genes associated to reactions described in the scaffold, we look for possible orthologs in the target organism. If certain conditions are met, the reaction is considered to be conserved, and added to the network of the target organism.

This method of projection can be applied to any pair of phylogenetically close species. Given a set of ortholog maps between two genomes, and a well-annotated metabolic model for one of them, it automatically produces a draft model for the target, providing a well-documented starting point for manual curation.

Well-curated models include information about the dependency of each reaction on proteins and genes, which is called Gene-Protein-Reaction associations (GPR). The Gene Association is the dependency of a reaction on the presence of a combination of genes, described as a logical formula between gene identifiers. For example, *S. cerevisiae* reaction R_0005 (“1,3-beta-glucan synthase”) can be performed by either the product of gene YLR342W (*FKS1*) or the product of gene YGR032W (*GSC2*), so its Gene Association is “(YGR032W or YLR342W)”.

During the reconstruction of the iNL895 *Y. lipolytica* model, we used three functional models published for *S. cerevisiae*: iMM904 [34], iIN800 [35] and the *consensus model* version 4.36 [36]. The latter was used as a scaffold for the reconstruction of the *Y. lipolytica* metabolic model, and will be referenced as the ‘scaffold model’ in what follows. We used the detailed fatty acid metabolism described in iIN800 [35] as a scaffold for *Y. lipolytica* fatty acid metabolism. From the scaffold model, we extracted the reactions predicted to be present in *Y. lipolytica*, the metabolites consumed and produced by them, the cellular compartments and all the non-enzymatic transport reactions. To make our model functional, we produced a list of genes that restored connectivity between the metabolites imported by the organism and the metabolic requirements of the biomass function. This list of genes provided as a starting point for the manual curation of the model.

### Orthology

Orthology detection based on sequence similarity is the most used approach to predict if a biological function, encoded by genes, is conserved between two organisms [37]. Some special cases need to be treated carefully: two ortholog genes, with originally similar functions, can mutate slightly and change its function, or can suffer a duplication, so only one of the two copies will keep the same biological function. Also, a fusion or fission event can integrate or divide certain domains into different genes. All those cases need to be integrated in the study of the conservation of function between two organisms and, in our experience, none of the current methods of ortholog mapping is good at all of them.

Based on homology between the genome of the scaffold (*S. cerevisiae*) and the genome of the target (*Y. lipolytica*), we determine if the original genes that encode the protein required for the enzymatic activity are conserved. Our method determines a) if a reaction is conserved, b) if a re-written gene association formula for the reaction is necessary ( Additional file 4: Figure S2).

For the reconstruction of the metabolic model of *Y. lipolytica*, we leveraged data provided by the Génolevures program [38], in the form of multi-species protein families and gene synteny. Protein families identify phylogenetic groups of proteins sequences that are a leading indication of functional analogy.

Génolevures protein families were further subdivided into groups with the same protein domain architecture (DOM), and synteny (SONS [38]) This initial high quality annotation allowed us to map most, but not all, of the genes used by the scaffold model, so we complemented this mapping with orthology from Inparanoid-DB [39] and OrthoMCL-DB [40].

In the cases of divergent predictions, consensus was determined by the following election procedure: From the different methods we produce a tally of the number of times each paralog group appears between all existing homolog map.

Our translator, using the rules described in Table 2, looks for the possible rewritings of the scaffold gene formulas in terms of genes of the target organism. To rewrite the new gene associations, an homolog map was built with the votes between all our available methods to detect orthologs ( Additional file 4: Figure S2).

**Table 2:** Gene association rewriting examples

Case	Reaction	Scaffold	Target
*M1* Gene loss $S_{1}\to \text{Ø}$	R_0490	YJR051W	–
*M2* Gene gain $\text{Ø}\to T_{1}$	R_2008	–	YALI0E34793g and YALI0D24431g
*M3* Two othologs $S_{1}\to T_{1}$	R_0240	YPL104W	YALI0F26433g
*M4* Duplication in scaffold $S_{1}\to T_{1}$, $S_{2}\to T_{1}$	R_1413	YEL006W or YIL006W	YALI0E16478g
*M5* Expansion in scaffold $S_{1k\;N}\to T_{1}$	R_0439	YIL009W or YMR246W or YOR317W	YALI0D17864g
*M6* Duplication in target $S_{1}\to \left(T_{1}\;or\;T_{2}\right)$	R_1551	YBL064C and **YCR083W**	YALI0F08195g and (**YALI0F01496g or YALI0E23540g**)
*M7* Expansion in target $S_{1}\to \left(T_{1}\;or\;T_{2}\;or\;K\;T_{N}\right)$	R_0415	**YGL205W** and YIL160C and YKR009C	YALI0E15378g and YALI0E18568g and **(YALI0E27654g or YALI0F10857g or YALI0C23859g or YALI0E32835g or YALI0E06567g or YALI0D24750g)**

Associations of genes to reactions in the model are useful for redundancy, and necessary for simulation of knockouts. When these associations are inherited from the scaffold, they must be rewritten to take into account expansion and contraction of protein families defined for homologous genes. The following examples illustrate the seven cases treated by the method.

The formulas that could not be resolved where reported to manual curation, as a possible loss of function (see Table 1). The resulting formulae were normalized to conjunctive normal form, as a list of alternative ways to encode the same reaction. Some examples of formula rewriting are provided in Table 2.

### A projected model

After rewriting gene associations, we kept the corresponding molecular species with its identifiers and annotations. We kept all the relevant non-enzymatic transport reactions and compartments.

A model that is able to predict growth is called a *functional* model. To predict growth, a measure of the molecular requirements to create a copy of the organism should be provided, in the form of a *biomass* function. This is usually obtained by the analysis of the molecular contents of live cells [41].

The biomass function of the *S. cerevisiae* model was used as a starting point for the *Y. lipolytica* model. Some coefficients were adjusted using the amount of DNA to be produced and the GC contents of the target organism [15]. G + C content and genome length of *Y. lipolytica* were obtained from the Génolevures program [38].

Automatic reconstructions may produce incomplete networks, missing the presence of some reactions that are part of an existing path of reactions. These “gaps” may lead to incorrect predictions, so they need to be fixed. We analyzed those gaps, generated lists of candidates, and included them as part of the manual curation stage. We also verified whether any of the *Y. lipolytica* genes were annotated with an EC code not present in the draft, adding new reactions to the model (see also Additional file 5).

**Figure 1:**
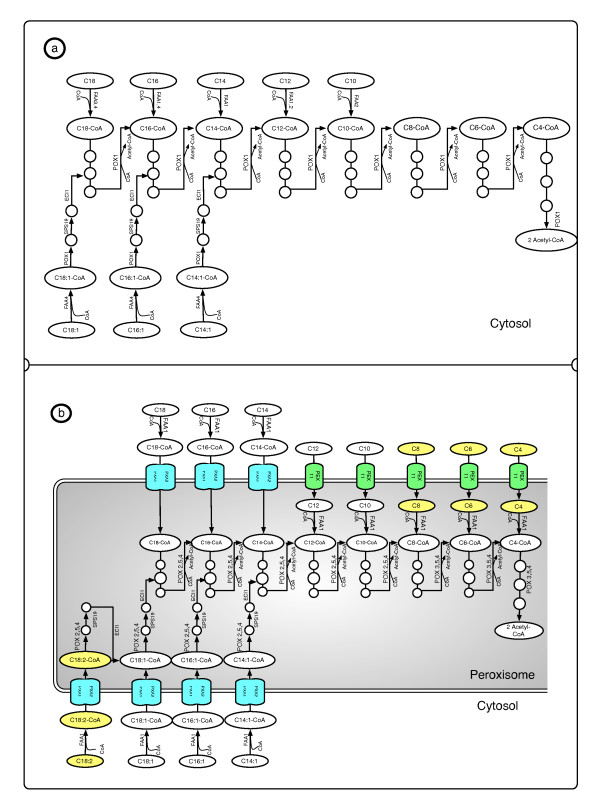
**Projecting Fatty Acid *β* -oxidation from S. cerevisiaeto Y. lipolytica. ** This simplified schematic view shows how the Fatty Acid *β*-oxidation scaffold pathway from *S. cerevisiae* iIN800 [35] was modified to adequately describe *Y. lipolytica*metabolism. (**a**) Simplified version of fatty acid *β*-oxidation diagram of *S. cerevisiae* iIN800. (**b**) Fatty acid *β*-oxidation in the reconstructed model for *Y. lipolytica*, with a constitutive peroxisome compartment and cytosol ↔ peroxisome transport reactions. Species-specific transport mechanisms for long and short fatty acid chains (*PXA1,2* and *PEX11*) are highlighted in *green* and *blue*. Long chains are activated (-CoA) before being transported to the peroxisome. *Y. lipolytica* can directly process Octanoic (C8), Hexanoic (C6), Butyric (C4) acid, and C18:2, so they were added to our model (in *yellow*). Our method predicted the family expansion of *S. cerevisiae POX1/FOX1* into *POX1-6*, and the reduction of *S. cerevisiae* family *FAA1-4* to *FAA1* (YALI0D17864g), which modified the genome associations of most of the pathway. *POX1-6* are written in order of specificity: *POX2,5,4* for long chains and *POX3,5,4* for short chains [42]

Given the importance of compartmentalization in eukaryotic organism, we built a model with 16 compartments, allowing us to map reactions and metabolites to different parts of the cell. We are interested in the oleaginous nature of *Y. lipolytica*, and its possible biotechnological applications, so it was critical to focus on the differences in fatty acid metabolism with respect to other yeasts. We started with the description of *β*-oxydation and fatty acid elongation from iIN800, projected them to *Y. lipolytica*, and manually modified to mirror the relevant literature (Figure 1).

We used the diagram of iIN800 [35] as an starting point for our own diagram of *Y. lipolytica* metabolism. This poster was used to discuss the draft model with the curators, who suggested changes based on their experience with the modeled species. These changes were translated to edits operations, and applied to our draft model.

The feedback obtained from the simulations of growth under different conditions (see below) and the results of gap-filling analysis were also used as part of the manual curation.

### Validation

To assess the predictive power of our metabolic model, we compared growth predictions, obtained using Flux Balance Analysis (FBA) [14], against 152 experimental results extracted from the literature. The effects of media conditions on growth, and the effects of gene knockouts in the system were included as constraints to the linear programming problem solved during FBA. From the literature we manually extracted experimental evidence (a growth/no growth indicator or a growth curve in time for each condition/deletion).

When growth curves were provided, we calculated a boolean value representing growth (true) or no growth (false), where the threshold was decided based on 1/3 of the average of growth in time (OD), for all mutants studied [43,44]. The same was done with simulated results: a threshold was used to decide between growth and no growth.

A confusion matrix and geometric mean [45] was used to measure the accuracy of our predictions versus experimental results. This approach was used to assess the quality of a model as a predictor, as it was done with the reconstruction of *S. cerevisiae* iIN800 [35] and iLL672 [46].

From the list of experimental results from the literature we produced a table of experiments, summarizing media conditions, gene knockouts, and observed growth (See Additional file 2: Table S2).

The description of media conditions were not standard between different works, so we defined, to the best of our knowledge, a base condition based on YPD, where only non-carbon sources were available (nitrogen, oxygen, etc.). This was modified for each simulation, controlling the availability of different carbon sources. The name of media conditions used in Additional file 2: Table S2, were obtained from the literature listed in Table 1, and describe the following combinations: YNBD: base + Glucose, YNBcas: YNBD + Casaminoacids, YNBO: base + Oleic acid, YNBC10: base + Decane, YNBC16: base + Hexadecane, YNBT: base + Trybutirin, YNBDptr: YNBD + Putrescine, YNBDtry: YNBD + Tryptophane.

We used FBA from COBRA Tools [47] to predict growth rate under different media conditions and gene knockouts that matched the available experimental results. From those simulations and the associated experiment, we automatically generated MATLAB tests, which generated an accuracy report of our model, consisting of False Positives/Negatives, True Positives/Negatives between the expected and predicted phenotype. The MATLAB file that simulates the 152 experiments is available as Additional file 6. The results are also available in Additional file 2: Table S2.

We called our reconstructed model iNL895, following the rules defined in [48]. We produced a version of our model in SBML format (Systems Biology Markup Language) [49], in order to analyze it with compatible existing tools, and share it with the community (see Additional file 7). An updated COBRA-compatible SBML version of our model can be retrieved from the BioModels database (http://biomodels.org), searching for the model id MODEL1111190000.

## Competing interests

The authors declare that they have no competing interests.

## Authors’ contributions

NL and DJS conceived the study and wrote the paper. NL implemented the reconstruction method and produced the model. TD and JMN provided expert knowledge in the form of manual curation. All authors read and approved the final manuscript.

## Supplementary Material

Additional file 1 Table S1. Manual curation of lost reactions. In many cases, orthology results fail to associate a target gene to an enzyme-coding gene in the scaffold model, suggesting that the reaction is absent. Each of these predictions were manually reviewed, where a reaction was confirmed as being absent (‘Lost’), or was upheld (‘Retained’) when empirical evidence was available. Genes for which no ortholog could be found are underlined in the gene association column. Click here for file

Additional file 2 Table S2. Validation of the iNL895 model. This table lists 152 experiments extracted from the literature, detailing media conditions, gene KOs, and observed growth (as yes/no). It also includes our simulations of the same experiments, obtained using FBA/COBRA Tools, and the comparison between observed and the simulated growth. Click here for file

Additional file 3 Figure S1. Projection pipeline from S. cerevisiae scaffold model to *Y. lipolytica* iNL895. The three main parts of our pipeline for the reconstruction of the *Y. lipolytica* model are: Projection, where the *S. cerevisiae* scaffold model and the information from different sources of orthology between *S. cerevisiae* and *Y. lipolytica* are used to produce a draft model, Curation, where the expert curators revised the candidates for gap-filling and added species-specific reactions and Validation, where experiments obtained from the literature were compared with our simulations, producing a detailed accuracy report. Click here for file

Additional file 4 Figure S2. Gene Association rewrite from *S. cerevisiae* reactions to *Y. lipolytica*. Pipeline for gene-association rewriting, as part of the projection of *Y. lipolytica* iNL895 model. From the 4 ortholog maps provided by different methods, a map of votes of possible ortholog mappings is created. Then, from the scaffold model, we extracted gene associations for each reaction, and re-wrote them based on our map of homologs (e.g.: Reaction1: (SourceGene1 or SourceGene2) ↔ (TargetGene1)). The new reactions, this time associated with *Y. lipolytica* genes, constituted the base of the reconstructed model. Click here for file

Additional file 5 **Selected gene annotations in *Y. lipolytica* **. This table lists *Y. lipolytica* genes used in the manual curation of the metabolic model. Click here for file

Additional file 6 **Complete validation tests for *Y. lipolytica.* ** This MATLAB file runs the validation tests of the *Y. lipolytica* metabolic model. It requires the COBRA Toolbox (2.0+). Each of the 152 tests is declared as a MATLAB function, in order to help the curation process. All tests can be ran in batch mode using: matlab -nodisplay -nosplash -nojvm -r “model0=runTests(‘supp_2.xml’, ‘test.results’); exit;” Click here for file

Additional file 7 ** *Y. lipolytica* iNL895 SBML model.** SBML representation of the reconstructed model of *Y. lipolytica*. This XML file is compatible with SBML Level 2, Version 4, and has been tested with COBRA Toolbox (2.0) and CellDesigner (4.1). This model can also be retrieved from the BioModels database (http://biomodels.org), under model id MODEL1111190000. Click here for file
