# Supplementary material for: A genome-scale metabolic model of the lipid-accumulating yeast Yarrowia lipolytica
Source: BMC Syst Biol. 2012 May 4;6:35. doi: 10.1186/1752-0509-6-35 (PMC3443063; doi:10.1186/1752-0509-6-35)
Supplement: Additional file 1 Table S1. — Manual curation of lost reactions. In many cases, orthology results fail to associate a target gene to an enzyme-coding gene in the scaffold model, suggesting that the reaction is absent. Each of these predictions were manually reviewed, where a reaction was confirmed as being absent (‘Lost’), or was upheld (‘Retained’) when empirical evidence was available. Genes for which no ortholog could be found are underlined in the gene association column. [file 1752-0509-6-35-S1.doc]

## Supplementary Table 1 - Manual curation of lost reactions

In many cases, orthology results fail to associate a target gene to an enzyme-coding gene in the scaffold model, suggesting that the reaction is absent. Each of these predictions were manually reviewed, where a reaction was confirmed as being absent (‘Lost’), or was upheld (‘Retained’) when empirical evidence was available. Genes for which no ortholog could be found are underlined in the gene association column.

| Scaffold reactions | Reaction Name | Reaction Gene Association | Reaction Status | Notes |
| --- | --- | --- | --- | --- |
| Continued on next page | | | | |
| R_0246 | Mitochondrial ATP synthase | Q0080 and Q0085 and Q0130 and YBL099W and YBR039W and YDL004W and YDR298C and YDR377W and YKL016C and YLR295C and YJR121W and YML081C-A and YPL078C and YPL271W and (YDR322C-A or YPR020W) | Retained | Only epsilon subunit lost |
| R_0750 …R_0755 | NatC acetylation | (YCR020C-A and YEL053C and YPR051W) | Retained | Reaction probably exists, unconnected to the rest of the network |
| R_0249 | ATPase, cytosolic | (YCR024C-A and YEL017C-A and YGL008C) | Retained | Not conserved genes are regulators of conserved gene. |
| R_0369 | dethiobiotin synthase | YNR057C | Retained | no gene ortholog, reaction should exists (there is biotin production) |
| R_29_bh …R_36_bh | MIPC synthase | (YBR036C and YPL057C) or (YBR036C and YBR161W) | Lost | Lost sphingolipid metabolism |
| R_0216 | α-glucosidase | (YBR299W or YGR287C or YGR292W) | Lost | No growth on sucrose by *Y. lipolytica* |
| R_0528 | glycerol-3-phosphatase | (YER062C or YIL053W) | Lost | Lack of reaction probably favors lipid accumulation |
| R_0176 …R_0180 | alcohol acetyltransferase (2-methylbutanol, ethanol, isoamyl alcohol, isobutyl, phenylethanol) | (YGR177C or YOR377W) | Lost | No alcohol production by *Y. lipolytica* |
| R_0653 | L-asparaginase | (YLR155C or YLR157C or YLR158C or YLR160C) | Lost | *S. cerevisiae*specific |
| R_37_bh …R_46_bh | inositol-phospho-transferase | YDR072C | Lost | Not enough info to decide |
| R_0652 | L-asparaginase | YDR321W | Lost | Alternative pathway exist |
| R_0670 | L-tyrosine N-formyltransferase | YDR403W | Lost | Confirmed lost |
| R_0489, R_0980 | fumarate reductase FMN | YEL047C | Lost | No anaerobic growth by *Y. lipolytica* |
| R_0490 | fumarate reductase FMN | YJR051W | Lost | No anaerobic growth by *Y. lipolytica* |
| R_0474, R_0475 | FMN reductase | YLR011W | Lost | Not enough info to decide |
| R_0062, R_1033 | (3-isopropyl-malate, trans-aconitate) 3-methyltransferase | YER175C | Lost | Deleted in sequenced strain of *Y. lipolytica*E150 (*leu2-270*) |
| R_0397 | endopoly-galacturonase | YJR153W | Lost | present in only few hemiascomycetous yeasts |
| R_1336 | iron (II) transport | YMR319C | Lost | present in only few hemiascomycetous yeasts |
| R_0153, R_0155, R_0359 | (adenine, adenosine, deoxyadenosine) deaminase | YNL141W | Lost | Alternative pathway exist |
| R_0226 | argininosuccinate synthase | YOL058W | Lost | Alternative pathway exist |
| R_0134 | acyl carrier protein synthase | YPL148C | Lost | No ortholog, probably highly divergent |
| R_3_bh, R_4_bh, R_8_bh, R_9_bh | ceramide synthase | (YHL003C or YHL008C) and YMR298W | Lost | Lost sphingolipid metabolism |
