# Supplementary material for: A genome-scale metabolic model of the lipid-accumulating yeast Yarrowia lipolytica
Source: BMC Syst Biol. 2012 May 4;6:35. doi: 10.1186/1752-0509-6-35 (PMC3443063; doi:10.1186/1752-0509-6-35)
Supplement: Additional file 2 Table S2. — Validation of the iNL895 model. This table lists 152 experiments extracted from the literature, detailing media conditions, gene KOs, and observed growth (as yes/no). It also includes our simulations of the same experiments, obtained using FBA/COBRA Tools, and the comparison between observed and the simulated growth. [file 1752-0509-6-35-S2.pdf]

### Additional Table 1 - Validation of the Y. lipolytica model

Validation of the *Y. lipolytica* model with respect to experimental evidence of *Y. lipolytica* growth under different media conditions and gene knockouts. Expected Growth was obtained by the referenced literature. Simulated Growth was obtained using flux balance analysis (FBA), optimizing over the biomass function of our model, and converted to a qualitative phenotype/no phenotype value by thresholding. In both cases, the symbols represent '+': growth; '–':no growth; 'n/a': condition cannot be simulated with current model, but are provided for future model improvements. The "Result" column compares the two: TP/FP: True/False Positives, TN/FN: True/False Negatives. The simulated models shows a sensitivity of 0.68, a specificity of 0.61 and an accuracy (geometric mean between sensitivity and specificity) of 0.65.

| Ref | Media | *Y. lipolytica* knocked locus | *S. cerevisiae* ortholog | Gene name | Exp. Growth | Simul. Growth | Result |
|---|---|---|---|---|---|---|---|
| [33] | YNBO | YALI0C06347g | YGL124C | *MON1* | + | n/a | n/a |
| [33] | YNBC16 | YALI0C06347g | YGL124C | *MON1* | + | n/a | n/a |
| [33] | YNBT | YALI0C06347g | YGL124C | *MON1* | + | n/a | n/a |
| [33] | YNBD | YALI0D27126g | YDR353W<br>YHR106W | *TRR1*<br>*TRR2* | + | - | FN |
| [33] | YNBD | YALI0E14729g | YOR153W | *PDR5* | + | + | TP |
| [33] | YNBO | YALI0E14729g | YOR153W | *PDR5* | + | - | FN |
| [33] | YNBC10 | YALI0E14729g | YOR153W | *PDR5* | + | + | TP |
| [33] | YNBC16 | YALI0E14729g | YOR153W | *PDR5* | - | - | TN |
| [33] | YNBT | YALI0E14729g | YOR153W | *PDR5* | + | - | FN |
| [33] | YNBD | YALI0E09405g | YGL153W | *PEX14* | + | n/a | n/a |
| [33] | YNBO | YALI0E09405g | YGL153W | *PEX14* | - | n/a | n/a |
| [33] | YNBC10 | YALI0E09405g | YGL153W | *PEX14* | - | n/a | n/a |
| [33] | YNBC16 | YALI0E09405g | YGL153W | *PEX14* | - | n/a | n/a |
| [33] | YNBD | YALI0D26081g | YLL051C<br>YOL152W<br>YOR381W<br>YOR384W | *FRE3-7* | + | n/a | n/a |
| [33] | YNBO | YALI0D26081g | YLL051C<br>YOL152W<br>YOR381W<br>YOR384W | *FRE3-7* | + | n/a | n/a |
| [33] | YNBC10 | YALI0D26081g | YLL051C<br>YOL152W<br>YOR381W<br>YOR384W | *FRE3-7* | - | n/a | n/a |
| [33] | YNBC16 | YALI0D26081g | YLL051C<br>YOL152W<br>YOR381W<br>YOR384W | *FRE3-7* | + | n/a | n/a |
| [33] | YNBT | YALI0D26081g | YLL051C<br>YOL152W<br>YOR381W<br>YOR384W | *FRE3-7* | + | n/a | n/a |
| [33] | YNBD | YALI0F04095g | YDL066W | *IDP1* | - | + | FP |
| [33] | YNBO | YALI0F04095g | YDL066W | *IDP1* | - | - | TN |
| [33] | YNBT | YALI0F04095g | YDL066W | *IDP1* | - | - | TN |
| [33] | YNBD | YALI0E09405g | YGL153W | *PEX14* | + | n/a | n/a |
| [33] | YNBO | YALI0E09405g | YGL153W | *PEX14* | - | n/a | n/a |
| [33] | YNBC10 | YALI0E09405g | YGL153W | *PEX14* | - | n/a | n/a |
| [33] | YNBC16 | YALI0E09405g | YGL153W | *PEX14* | - | n/a | n/a |
| [33] | YNBT | YALI0E09405g | YGL153W | *PEX14* | + | n/a | n/a |
| [33] | YNBD | YALI0C21582g | YGL059W | *PKP2* | + | n/a | n/a |
| [33] | YNBO | YALI0C21582g | YGL059W | *PKP2* | - | n/a | n/a |
| [33] | YNBC10 | YALI0C21582g | YGL059W | *PKP2* | - | n/a | n/a |
| [33] | YNBC16 | YALI0C21582g | YGL059W | *PKP2* | + | n/a | n/a |

Continued on next page

| Ref | Media | *Y. lipolytica* knocked locus | *S. cerevisiae* ortholog | Gene name | Exp. Growth | Simul. Growth | Result |
|---|---|---|---|---|---|---|---|
| [33] | YNBD | YALI0E34672g | YJR095W | *ACR1* | + | + | TP |
| [33] | YNBO | YALI0E34672g | YJR095W | *ACR1* | - | - | TN |
| [33] | YNBC10 | YALI0E34672g | YJR095W | *ACR1* | - | + | FP |
| [33] | YNBC16 | YALI0E34672g | YJR095W | *ACR1* | - | - | TN |
| [33] | YNBT | YALI0E34672g | YJR095W | *ACR1* | - | - | TN |
| [33] | YNBD | YALI0B13970g | YIL155C | *GUT2* | + | + | TP |
| [33] | YNBC10 | YALI0B13970g | YIL155C | *GUT2* | + | + | TP |
| [33] | YNBC16 | YALI0B13970g | YIL155C | *GUT2* | + | - | FN |
| [33] | YNBD | YALI0E06831g | | *PEX20* | + | n/a | n/a |
| [33] | YNBO | YALI0E06831g | | *PEX20* | - | n/a | n/a |
| [33] | YNBC10 | YALI0E06831g | | *PEX20* | - | n/a | n/a |
| [33] | YNBC16 | YALI0E06831g | | *PEX20* | - | n/a | n/a |
| [33] | YNBT | YALI0E06831g | | *PEX20* | + | n/a | n/a |
| [33] | YNBD | YALI0F18216g | YFL001W | *DEG1* | + | + | TP |
| [33] | YNBC16 | YALI0F18216g | YFL001W | *DEG1* | + | - | FN |
| [33] | YNBT | YALI0F18216g | YFL001W | *DEG1* | + | - | FN |
| [33] | YNBD | YALI0E03058g | YPR128C | *PMP34* | + | + | TP |
| [33] | YNBO | YALI0E03058g | YPR128C | *PMP34* | + | - | FN |
| [33] | YNBC10 | YALI0E03058g | YPR128C | *PMP34* | - | + | FP |
| [33] | YNBC16 | YALI0E03058g | YPR128C | *PMP34* | + | - | FN |
| [33] | YNBT | YALI0E03058g | YPR128C | *PMP34* | + | - | FN |
| [33] | YNBD | YALI0C16885g | YER065C | *ICL1* | + | + | TP |
| [33] | YNBO | YALI0C16885g | YER065C | *ICL1* | - | - | TN |
| [33] | YNBC10 | YALI0C16885g | YER065C | *ICL1* | - | - | TN |
| [33] | YNBC16 | YALI0C16885g | YER065C | *ICL1* | - | - | TN |
| [33] | YNBT | YALI0C16885g | YER065C | *ICL1* | - | - | TN |
| [33] | YNBD | YALI0E14729g | YOL075C YNR070W YDR011W | *PDR5* | + | + | TP |
| [33] | YNBO | YALI0E14729g | YOL075C YNR070W YDR011W | *PDR5* | + | - | FN |
| [33] | YNBC10 | YALI0E14729g | YOL075C YNR070W YDR011W | *PDR5* | + | + | TP |
| [33] | YNBC16 | YALI0E14729g | YOL075C YNR070W YDR011W | *PDR5* | - | - | TN |
| [33] | YNBT | YALI0E14729g | YOL075C YNR070W YDR011W | *PDR5* | + | - | FN |
| [34] | Lactose | n/a, media only | | | - | n/a | n/a |
| [34] | D-Galactose | n/a, media only | | | - | + | FP |
| [35] | YNBD | YALI0A15972g | YLR377C | *FBP1* | + | + | TP |
| [35] | Ethanol | YALI0A15972g | YLR377C | *FBP1* | + | - | FN |
| [35] | Glycerol | YALI0A15972g | YLR377C | *FBP1* | + | - | FN |

Continued on next page

| Ref | Media | *Y. lipolytica* knocked locus | *S. cerevisiae* ortholog | Gene name | Exp. Growth | Simul. Growth | Result |
|---|---|---|---|---|---|---|---|
| [35] | Acetate | YALI0A15972g | YLR377C | *FBP1* | + | - | FN |
| [36] | YNBD | YALI0C24101g | YGL062W | *PYC1* | + | + | TP |
| [36] | Ethanol | YALI0C24101g | YGL062W | *PYC1* | + | + | TP |
| [36] | Aspartate | YALI0C24101g | YGL062W | *PYC1* | + | + | TP |
| [36] | Glutamate | YALI0C24101g | YGL062W | *PYC1* | + | + | TP |
| [36] | YNBD | YALI0C16885g | YER065C | *ICL1* | + | + | TP |
| [36] | Ethanol | YALI0C16885g | YER065C | *ICL1* | - | - | TN |
| [36] | Aspartate | YALI0C16885g | YER065C | *ICL1* | + | + | TP |
| [36] | Glutamate | YALI0C16885g | YER065C | *ICL1* | + | + | TP |
| [36] | YNBD | YALI0C24101g<br>YALI0C16885g | YGL062W<br>YER065C | *ICL1*<br>*PYC1* | - | + | FP |
| [36] | Ethanol | YALI0C24101g<br>YALI0C16885g | YGL062W<br>YER065C | *ICL1*<br>*PYC1* | - | - | TN |
| [36] | Aspartate | YALI0C24101g<br>YALI0C16885g | YGL062W<br>YER065C | *ICL1*<br>*PYC1* | + | + | TP |
| [36] | Glutamate | YALI0C24101g<br>YALI0C16885g | YGL062W<br>YER065C | *ICL1*<br>*PYC1* | + | + | TP |
| [37] | YNBC10 | YALI0E19514g | YCR077C | *PAT1* | - | n/a | n/a |
| [37] | YNBD | YALI0E19514g | YCR077C | *PAT1* | + | n/a | n/a |
| [37] | Glycerol | YALI0E19514g | YCR077C | *PAT1* | + | n/a | n/a |
| [38] | YNBD | YALI0D24431g<br>YALI0E34793g | | *ACL1* | + | + | TP |
| [38] | YNBO | YALI0D24431g<br>YALI0E34793g | | *ACL1* | + | - | FN |
| [39] | Acetate | YALI0C16885g | YER065C | *ICL1* | - | - | TN |
| [39] | YNBO | YALI0C16885g | YER065C | *ICL1* | - | - | TN |
| [39] | YNBD | YALI0C16885g | YER065C | *ICL1* | + | + | TP |
| [39] | Acetate | YALI0E15708g | YNL117W | *MLS1* | + | + | TP |
| [39] | YNBO | YALI0E15708g | YNL117W | *MLS1* | + | - | FN |
| [39] | YNBD | YALI0E15708g | YNL117W | *MLS1* | + | + | TP |
| [39] | Acetate | YALI0E02684g | YCR005C<br>YNR001C | *CIT2* | + | + | TP |
| [39] | YNBO | YALI0E02684g | YCR005C<br>YNR001C | *CIT2* | + | - | FN |
| [39] | YNBD | YALI0E02684g | YCR005C<br>YNR001C | *CIT2* | + | + | TP |
| [40] | YNBD | YALI0B13970g | YIL155C | *GUT2* | + | + | TP |
| [40] | Glycerol | YALI0B13970g | YIL155C | *GUT2* | - | + | FP |
| [40] | YNBO | YALI0B13970g | YIL155C | *GUT2* | + | - | FN |
| [40] | YNBD | YALI0B13970g<br>YALI0E32835g<br>YALI0F10857g<br>YALI0D24750g<br>YALI0E27654g<br>YALI0C23859g<br>YALI0E06567g | YIL155C<br>YGL205W | *GUT2*<br>*POX1*<br>*POX2*<br>*POX3*<br>*POX4*<br>*POX5*<br>*POX6* | + | + | TP |

Continued on next page

| Ref | Media | *Y. lipolytica* knocked locus | *S. cerevisiae* ortholog | Gene name | Exp. Growth | Simul. Growth | Result |
|---|---|---|---|---|---|---|---|
| [40] | Glycerol | YALI0B13970g<br>YALI0E32835g<br>YALI0F10857g<br>YALI0D24750g<br>YALI0E27654g<br>YALI0C23859g<br>YALI0E06567g | YIL155C<br>YGL205W | *GUT2*<br>*POX1*<br>*POX2*<br>*POX3*<br>*POX4*<br>*POX5*<br>*POX6* | - | + | FP |
| [40] | YNBO | YALI0B13970g<br>YALI0E32835g<br>YALI0F10857g<br>YALI0D24750g<br>YALI0E27654g<br>YALI0C23859g<br>YALI0E06567g | YIL155C<br>YGL205W | *GUT2*<br>*POX1*<br>*POX2*<br>*POX3*<br>*POX4*<br>*POX5*<br>*POX6* | - | - | TN |
| [40] | YNBD | YALI0E32835g<br>YALI0F10857g<br>YALI0D24750g<br>YALI0E27654g<br>YALI0C23859g<br>YALI0E06567g | YIL155C<br>YGL205W | *POX1*<br>*POX2*<br>*POX3*<br>*POX4*<br>*POX5*<br>*POX6* | + | + | TP |
| [40] | Glycerol | YALI0E32835g<br>YALI0F10857g<br>YALI0D24750g<br>YALI0E27654g<br>YALI0C23859g<br>YALI0E06567g | YIL155C<br>YGL205W | *POX1*<br>*POX2*<br>*POX3*<br>*POX4*<br>*POX5*<br>*POX6* | + | + | TP |
| [40] | YNBO | YALI0E32835g<br>YALI0F10857g<br>YALI0D24750g<br>YALI0E27654g<br>YALI0C23859g<br>YALI0E06567g | YIL155C<br>YGL205W | *POX1*<br>*POX2*<br>*POX3*<br>*POX4*<br>*POX5*<br>*POX6* | - | - | TN |
| [41] | YNBD | YALI0D02629g | YOR222W<br>YPL134C | *ODC1* | - | + | FP |
| [41] | YNBD+ putrescine | YALI0D02629g | YOR222W<br>YPL134C | *ODC1* | + | + | TP |
| [42] | YNBD | YALI0B07667g | YDR007W | *TRP1* | - | + | FP |
| [42] | YNBD+ tryptophane | YALI0B07667g | YDR007W | *TRP1* | + | + | TP |
| [32] | 2-Keto-D-Gluconate | n/a, media only | | | - | n/a | n/a |
| [32] | a,a-Trehalose | n/a, media only | | | - | + | FP |
| [32] | Arbutin | n/a, media only | | | - | n/a | n/a |
| [32] | Butane 2,3 diol | n/a, media only | | | - | n/a | n/a |
| [32] | Cellobiose | n/a, media only | | | - | n/a | n/a |
| [32] | Citrate | n/a, media only | | | + | + | TP |
| [32] | D-Arabinose | n/a, media only | | | - | - | TN |

Continued on next page

| Ref | Media | *Y. lipolytica* knocked locus | *S. cerevisiae* ortholog | Gene name | Exp. Growth | Simul. Growth | Result |
|---|---|---|---|---|---|---|---|
| [32] | D-Galactonate | n/a, media only | | | - | n/a | n/a |
| [32] | D-Galactose | n/a, media only | | | - | + | FP |
| [32] | D-Galacturonate | n/a, media only | | | - | - | TN |
| [32] | D-glucarate | n/a, media only | | | - | n/a | n/a |
| [32] | D-Glucitol | n/a, media only | | | + | + | TP |
| [32] | D-Gluconate | n/a, media only | | | + | n/a | n/a |
| [32] | D-Glucono-1,5-lactone | n/a, media only | | | + | n/a | n/a |
| [32] | D-Glucosamine | n/a, media only | | | - | + | FP |
| [32] | D-Glucose | n/a, media only | | | + | + | TP |
| [32] | D-Glucuronate | n/a, media only | | | - | n/a | n/a |
| [32] | D-Mannitol | n/a, media only | | | + | n/a | n/a |
| [32] | D-Ribose | n/a, media only | | | - | + | FP |
| [32] | D-Xylose | n/a, media only | | | - | + | FP |
| [32] | DL-Lactate | n/a, media only | | | + | + | TP |
| [32] | Erythritol | n/a, media only | | | + | n/a | n/a |
| [32] | Ethanol | n/a, media only | | | + | + | TP |
| [32] | Galactitol | n/a, media only | | | - | n/a | n/a |
| [32] | Glycerol | n/a, media only | | | + | + | TP |
| [32] | Inulin | n/a, media only | | | - | n/a | n/a |
| [32] | L-Arabinitol | n/a, media only | | | - | - | TN |
| [32] | L-Arabinose | n/a, media only | | | - | - | TN |
| [32] | L-Rhamnose | n/a, media only | | | - | n/a | n/a |
| [32] | L-Sorbose | n/a, media only | | | - | - | TN |
| [32] | Lactose | n/a, media only | | | - | n/a | n/a |
| [32] | Maltose | n/a, media only | | | - | - | TN |
| [32] | Me-a-D-Glucoside | n/a, media only | | | - | n/a | n/a |
| [32] | Melezitose | n/a, media only | | | - | n/a | n/a |
| [32] | Melibiose | n/a, media only | | | - | - | TN |
| [32] | Methanol | n/a, media only | | | - | n/a | n/a |
| [32] | myo-Inositol | n/a, media only | | | - | - | TN |
| [32] | Propane 1,2 diol | n/a, media only | | | - | n/a | n/a |
| [32] | Quinic acid | n/a, media only | | | - | n/a | n/a |
| [32] | Raffinose | n/a, media only | | | - | n/a | n/a |
| [32] | Ribitol | n/a, media only | | | - | n/a | n/a |
| [32] | Salicin | n/a, media only | | | - | n/a | n/a |
| [32] | Starch | n/a, media only | | | - | n/a | n/a |
| [32] | Succinate | n/a, media only | | | + | + | TP |
| [32] | Sucrose | n/a, media only | | | - | + | FP |
| [32] | Xylitol | n/a, media only | | | - | + | FP |
| Overall results: | | TP: 39, TN: 25, FN: 18, FP: 16; accuracy: 0.65 | | | | | |
