# Supplementary figures and images for: A genome-scale metabolic model of the lipid-accumulating yeast Yarrowia lipolytica
Source: BMC Syst Biol. 2012 May 4;6:35. doi: 10.1186/1752-0509-6-35 (PMC3443063; doi:10.1186/1752-0509-6-35)

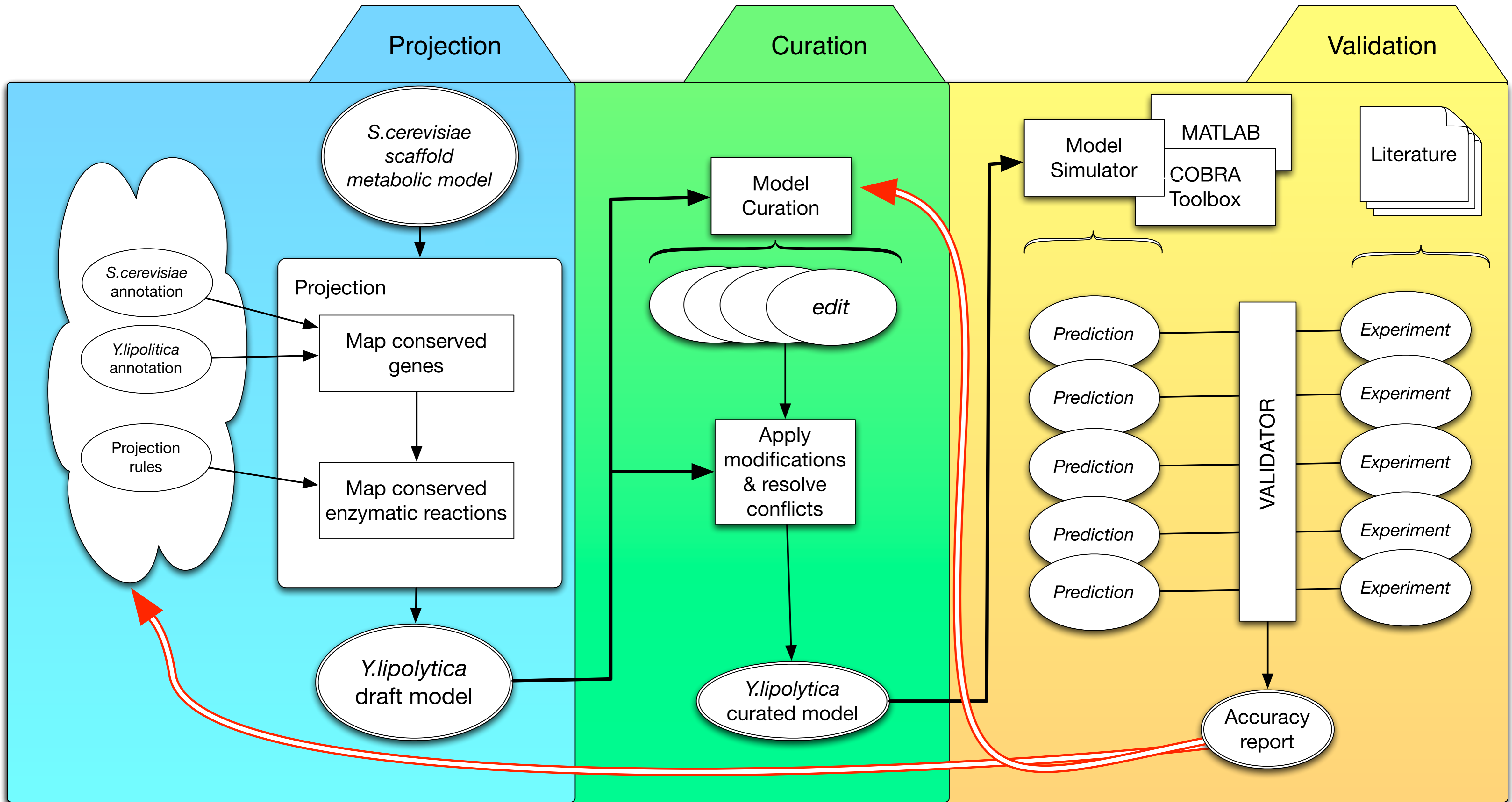

Supplement: Additional file 3 Figure S1. — Projection pipeline from S. cerevisiae scaffold model to Y. lipolytica iNL895. The three main parts of our pipeline for the reconstruction of the Y. lipolytica model are: Projection, where the S. cerevisiae scaffold model and the information from different sources of orthology between S. cerevisiae and Y. lipolytica are used to produce a draft model, Curation, where the expert curators revised the candidates for gap-filling and added species-specific reactions and Validation, where experiments obtained from the literature were compared with our simulations, producing a detailed accuracy report. [file 1752-0509-6-35-S3.pdf]

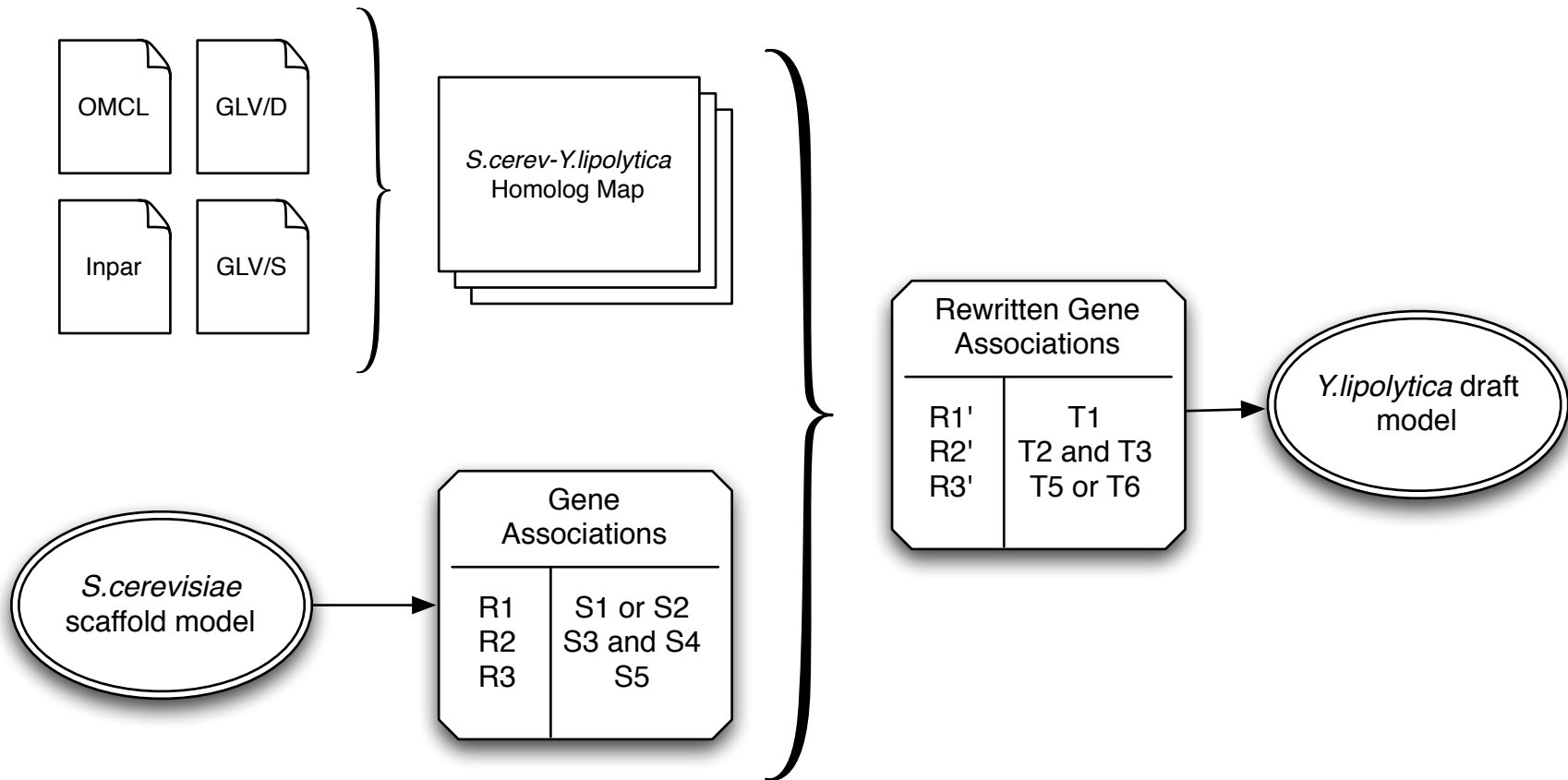

Supplement: Additional file 4 Figure S2. — Gene Association rewrite from S. cerevisiae reactions to Y. lipolytica. Pipeline for gene-association rewriting, as part of the projection of Y. lipolytica iNL895 model. From the 4 ortholog maps provided by different methods, a map of votes of possible ortholog mappings is created. Then, from the scaffold model, we extracted gene associations for each reaction, and re-wrote them based on our map of homologs (e.g.: Reaction1: (SourceGene1 or SourceGene2) ↔ (TargetGene1)). The new reactions, this time associated with Y. lipolytica genes, constituted the base of the reconstructed model. [file 1752-0509-6-35-S4.pdf]
